# Enhanced Detection and Recognition of Road Objects in Infrared Imaging Using Multi-Scale Self-Attention

**DOI:** 10.3390/s24165404

**Published:** 2024-08-21

**Authors:** Poyi Liu, Yunkang Zhang, Guanlun Guo, Jiale Ding

**Affiliations:** 1 School of Communication Engineering, Wuhan University of Technology, Wuhan 430070, China; 329057@whut.edu.cn (P.L.); 329334@whut.edu.cn (Y.Z.); 2 School of Automotive Engineering, Wuhan University of Technology, Wuhan 430070, China; 3 School of Safety and Emergency Management, Wuhan University of Technology, Wuhan 430070, China; 321579@whut.edu.cn

**Keywords:** infrared detection, self-attention mechanism, autonomous driving, YOLO algorithm, real-time processing

## Abstract

In infrared detection scenarios, detecting and recognizing low-contrast and small-sized targets has always been a challenge in the field of computer vision, particularly in complex road traffic environments. Traditional target detection methods usually perform poorly when processing infrared small targets, mainly due to their inability to effectively extract key features and the significant feature loss that occurs during feature transmission. To address these issues, this paper proposes a fast detection and recognition model based on a multi-scale self-attention mechanism, specifically for small road targets in infrared detection scenarios. We first introduce and improve the DyHead structure based on the YOLOv8 algorithm, which employs a multi-head self-attention mechanism to capture target features at various scales and enhance the model’s perception of small targets. Additionally, to prevent information loss during the feature transmission process via the FPN structure in traditional YOLO algorithms, this paper introduces and enhances the Gather-and-Distribute Mechanism. By computing dependencies between features using self-attention, it reallocates attention weights in the feature maps to highlight important features and suppress irrelevant information. These improvements significantly enhance the model’s capability to detect small targets. Moreover, to further increase detection speed, we pruned the network architecture to reduce computational complexity and parameter count, making the model suitable for real-time processing scenarios. Experiments on our self built infrared road traffic dataset (mainly including two types of targets: vehicles and people) show that compared with the baseline, our method achieves a 3.1% improvement in AP and a 2.5% increase in mAP on the VisDrone2019 dataset, showing significant enhancements in both detection accuracy and processing speed for small targets, with improved robustness and adaptability.

## 1. Introduction

With the rapid development of technology, autonomous driving technology has become a key direction for the future of transportation [1], attracting widespread attention globally [2]. According to predictions by the International Data Corporation (IDC), the number of autonomous vehicles worldwide will reach 5.4 million by 2025 [3]. However, safety concerns remain one of the main challenges hindering their large-scale commercial use. On the road, small objects such as pedestrians, cyclists, and small animals can pose threats to the safety of autonomous vehicles. According to statistics from the National Highway Traffic Safety Administration (NHTSA), in 2019, traffic accidents caused by undetected small objects accounted for 20% of all incidents. These accidents not only pose a threat to human life but also present significant challenges to the development of autonomous driving technology.

In modern autonomous driving technology, infrared imaging systems can operate under any weather and lighting conditions, especially in complex road environments, making infrared technology crucial for the rapid and accurate detection and recognition of small objects. Therefore, small target detection in infrared imagery has become a significant area of focus and research in the fields of computer vision and image processing. Infrared small targets typically refer to objects that are small in size, emit weak thermal radiation, and show little temperature difference from their surroundings, such as long-range missiles, drones, and motor vehicles [4]. Unlike visible light images, infrared images reflect the thermal radiation information of objects, which allows for the detection of objects over considerable distances in modern autonomous driving technology. This poses unique challenges. Firstly, small objects in infrared images often suffer from interference from complex backgrounds, low contrast, and noise, making it difficult to distinguish them from the background. Secondly, the small size of infrared targets, often just a few pixels, makes localization and recognition more challenging. Additionally, small targets in infrared images usually appear as hotspots or hot patches, with limited shape and texture information, increasing the difficulty of detection. To address these issues, researchers have proposed various methods for detecting small targets in infrared images. These methods include traditional image processing techniques such as filtering, thresholding, and morphological operations, as well as machine learning and deep learning approaches like support vector machines, convolutional neural networks, and recurrent neural networks. These methods have achieved certain successes in the field of infrared small target detection, such as their widespread application in infrared search and track systems, particularly in autonomous driving and military applications, including infrared guidance, early warning, air defense, and anti-missile systems.

For object detection methods, Lin et al. [5] proposed the RetinaNet object detection algorithm, which also incorporates the concept of multi-scale object detection. It uses ResNet (Residual Network) as the backbone to extract image features. To address the issue of class imbalance between positive and negative samples during model training, RetinaNet introduces a reshaped loss function, which not only improves detection speed but also enhances the effectiveness of small object detection. Similarly, Zhang et al. [6] proposed the MTCNN (Multi-task Convolutional Neural Network) algorithm, which employs multi-scale detection. Before feeding images into the network for training, the input images are scaled to different sizes, thereby enhancing the network’s robustness in detecting faces of varying sizes and significantly improving the accuracy of detecting small faces. Lin et al. [7] also introduced the Feature Pyramid Network (FPN). FPN generates multi-scale feature maps from the bottom up, and to combine low-resolution, strong semantic features with high-resolution, weak semantic features, it uses a top-down path and lateral connections. This structure allows for the full utilization of semantic information from feature maps at different scales, with minimal additional computational cost. Building on FPN, Liu et al. [8] proposed the PANet (Path Aggregation Network for Instance Segmentation), which achieved significant success in instance segmentation and also improved small object detection. In FPN, the shallow features lose considerable information after being passed through multiple layers in the bottom-up process. To address this, PANet adds a bottom-up path augmentation structure, which better preserves shallow feature information. Additionally, PANet introduces an adaptive feature pooling layer that aggregates candidate regions from each feature map, further enhancing feature fusion. Finally, a fully connected layer is used to capture different views of each candidate region, leading to better prediction results. Yan et al. [9] designed a local feature extraction module to segment feature maps and obtain local image features. They also developed a global feature extraction module to calculate the correlation between feature points, enriching the features used in the final prediction. Learnable weights were added to the feature layers involved in the final prediction to assist the model in detection. Moreover, the idea of feature map reuse was proposed to retain more information from high-dimensional feature maps. Comparative experiments on public datasets showed that the accuracy of the improved algorithm was significantly enhanced compared to the original algorithm.

In the field of infrared target detection, Feng et al. [10] established a typical dataset of infrared images for substation equipment and then optimized the learning rate in the YOLOv5 model using a dynamic decay method. They further optimized the model’s weight decay coefficient to prevent overfitting on the dataset. After two rounds of training, they successfully achieved the recognition and classification of typical substation equipment in infrared images. Their research not only addressed the issue of identifying and accurately classifying electrical equipment in substation infrared images but also provided a technical foundation for intelligent fault diagnosis of equipment based on substation infrared images.

Zhang [11] and colleagues proposed an efficient and accurate vehicle detection algorithm tailored for aerial infrared images, utilizing an enhanced YOLOv3 network. To improve detection efficiency, they designed a novel structure for the modified YOLOv3 network, reducing it to only 16 layers. They also expanded the anchor boxes to four scales to enhance the detection accuracy of small vehicles. Given the limited availability of infrared vehicle samples, they employed transfer learning to train the improved YOLOv3 network. The proposed algorithm was then evaluated using the VIVID and NPU datasets. Experimental results and in-depth analysis demonstrated that the algorithm achieved satisfactory and competitive vehicle detection performance.

Given the existing issues with infrared small target detection, such as low detection accuracy, poor real-time performance [12], and limited adaptability to complex scenarios [13], this study proposes a new method for detecting and recognizing infrared small targets by enhancing the YOLO model with a self-attention mechanism across multiple scales. This method aims to provide theoretical support and practical guidance for the development of autonomous driving technologies.The main innovations of this article are as follows:

1. Addressing the challenges of infrared small target detection, which is susceptible to background interference and typically suffers from low accuracy, this paper builds upon YOLOv8 by harnessing the efficient feature transmission capabilities of attention mechanisms. Through the use of self-attention across multiple scales, the model has been enhanced. To prevent model bloat, pruning techniques were employed to reduce the model’s parameter count. Experiments conducted on both proprietary and public datasets demonstrate that our model exhibits robustness and state-of-the-art performance.
2. Improvements across multiple scales: To address information loss in the traditional YOLO algorithm via the FPN structure, this work incorporates a model modification using a Gather-and-Distribute Mechanism constructed from self-attention. This significantly enhances the model’s ability to detect small objects. Additionally, the original detection head was improved by introducing a DyHead structure formed from multi-head self-attention, enabling the model to rapidly extract crucial information from the feature layers.Among them, * represents the operation between the feature map and the attention function
3. Model light-weighting operations: To facilitate better deployment of our model, we conducted further model pruning operations on the developed model. After pruning, the size of the model and the number of parameters were significantly reduced, while maintaining an acceptable level of accuracy loss. This process involves systematically removing less important parameters within the neural network, which not only reduces the computational burden but also improves the efficiency of the model. This optimization ensures that the model remains robust and agile, capable of performing effectively without compromising the essential detection capabilities.
4. Related experiments: The enhanced model was compared against different datasets and baseline models. Feature map visualization was employed to illustrate the improvements in model accuracy. The experimental results indicate that the proposed improved model has strong generalization capabilities. Our method achieves a 3.1% improvement in AP and a 2.5% increase in mAP on the VisDrone2019 dataset.

**Figure 1:**
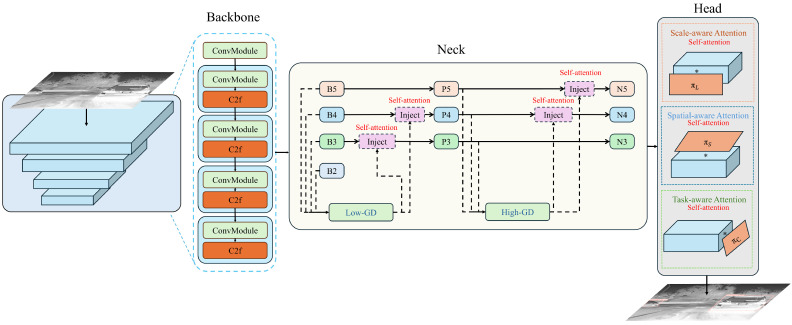
Framework diagram for improving the model.

The framework diagram of our proposed improved model is shown in Figure 1.

## 2. Related Work

### 2.1. Introduction to YOLO Algorithm

The You Only Look Once (YOLO) algorithm [14] is a groundbreaking single-stage object detection technique. It divides an image into multiple grids, predicts bounding boxes and their corresponding object categories within each grid, and uses Non-Maximum Suppression (NMS) to remove overlapping boxes. YOLOv1 is renowned for its quick detection speed; however, it is less effective for objects that are closely spaced or small. YOLOv2 [15], featuring the Darknet19 as its backbone network, adapts to various image sizes, improving small object detection accuracy. YOLOv3 [16] incorporated a Feature Pyramid Network (FPN) and Spatial Pyramid Pooling (SPP) modules, enhancing multi-scale detection and semantic information. YOLOv4 [17] increased accuracy by integrating the Mish activation function. YOLOv5 [18] introduced the C3 and SPPF modules, which enhanced feature robustness and detection performance. YOLOv7 [19] brought in a scalable efficient layer aggregation network (E-ELAN) with novel transition modules and re-parameterization techniques, boosting feature extraction and semantic expression, and further refining detection performance. The YOLO series has evolved to YOLOv9 [20], a testament to its enduring relevance as a single-stage detection algorithm. This paper concentrates on the widely adopted and stable YOLOv8 model, which not only enhances detection accuracy but also reduces computational demands, parameter count, and model size.

The advantages of the YOLO (You Only Look Once) algorithm in infrared detection scenarios are primarily reflected in the following aspects:

- Unlike traditional sliding window and region proposal network-based detection algorithms, YOLO transforms the object detection task into a regression problem, processing the entire image in one go. This significantly enhances the detection speed. According to recent research data, YOLO can process images at 45 frames per second, while the fastest algorithm in the R-CNN series [21], Fast R-CNN [22], processes only 7 frames per second. This high efficiency makes YOLO particularly suitable for scenarios where real-time response is crucial [23].
- As YOLO utilizes full image information during training, it adapts well to changes in the image [24]. Even in complex scenarios with varying lighting conditions or changes in object scale, YOLO maintains high detection accuracy [25].
- YOLO employs a global optimization strategy, considering the overall information of the image rather than just focusing on local features [26]. This capability enables YOLO to handle some challenges that traditional detection algorithms struggle with, such as detecting small or overlapping objects, with higher accuracy [27].
- The network structure of YOLO is simple, making it easy to integrate with other neural network structures [28]. For example, additional convolutional layers can be added to YOLO for feature extraction, or fully connected layers can be added for classification. This ease of integration allows YOLO to perform multiple tasks simultaneously in composite applications, such as pedestrian and vehicle detection in autonomous driving, while maintaining high processing speed and accuracy.

These advantages demonstrate the significant potential of YOLO for application in infrared detection scenarios. Its efficiency, accuracy, and robustness meet the complex environmental demands of autonomous driving, playing a vital role in advancing autonomous driving technologies.

### 2.2. Introduction to Attention Mechanisms

The attention mechanism, widely adopted in fields such as natural language processing, statistical learning, image detection, and speech recognition following the rapid development of deep learning, is a core technology that efficiently allocates information processing resources [29]. This concept is derived from studies of human attention and essentially involves focusing more on relevant segments of input while ignoring the less relevant ones. For example, when searching for human figures in an image, people tend to focus more on areas that match the characteristics of human figures and disregard areas that do not, effectively distributing attention where it’s most needed.

Attention is an indispensable, complex cognitive function in the human brain [30]. In daily life, people receive vast amounts of information through sight, hearing, and touch. Despite this barrage of external information, individuals can function in an orderly way because the human brain can intentionally or unintentionally select a small portion of this information for focused processing while ignoring the rest. This capability is known as attention.

The attention mechanism mimics this ability by assigning high weights to significant information and low weights to irrelevant details, continuously adjusting these weights to capture the most relevant information under varying circumstances [6]. This adaptability provides the mechanism with higher scalability and robustness.

**Figure 2:**
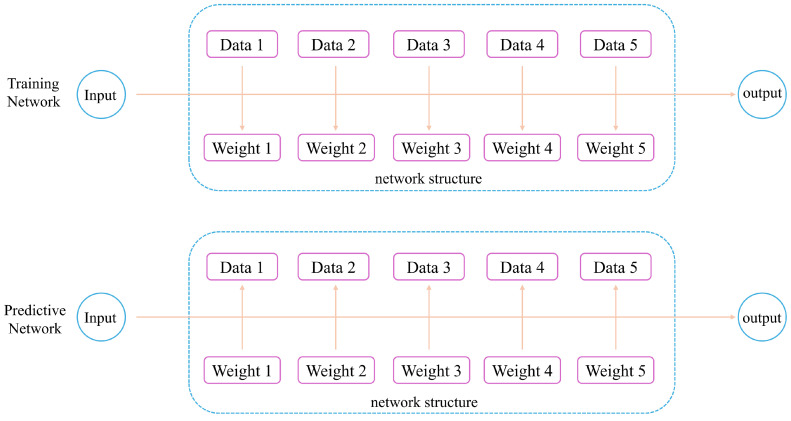
The basic framework of attention mechanism networks.

The basic framework of a network using attention mechanism is shown in Figure 2, illustrating how it prioritizes different parts of the input data, streamlining the process to enhance the model’s performance in tasks that require focused analysis.

The self-attention mechanism is an attention mechanism that computes attention within the sequence itself, assigning varying weights to different elements to capture internal relationships within the sequence [31]. In the context of infrared small target detection discussed in this article, a multi-head self-attention mechanism is used to improve detection accuracy by focusing on multiple subspaces of features.

In a multi-head self-attention mechanism, attention is calculated across multiple “heads”, with each head focusing on different parts of the feature space [32]. This method enables the model to consider information from various representational spaces at different positions [33], making it more robust and adaptable than single-headed attention. The self-attention mechanism functions by computing relationships between a query matrix *Q*, a key matrix *K*, and a value matrix *V*, using these relationships to concentrate on globally relevant information.

The core operation in self-attention is the Scaled Dot-product Attention (SDA), which is computed as follows:

1. Linear transformations: Each head in the multi-head attention mechanism performs separate linear transformations of the input *X* to create different sets of queries, keys, and values. This is performed using parameter matrices $W^{Q}$, $W^{K}$, and $W^{V}$, which are learned during training:
  (1) $$\;Q=XW^{Q},\;K=XW^{K},\;V=XW^{V}$$
  These matrices $W^{Q}$, $W^{K}$, and $W^{V}$ are unique to each head, allowing the model to learn different aspects of the data in each subspace.
2. Scaled Dot-product Attention: Each head computes the attention scores using the Scaled Dot-product Attention formula:
  (2) $$\;\mathrm{Attention}\;\left(Q,K,V\right)=\mathrm{softmax}\left(\frac{{\mathrm{QK}}^{T}}{\sqrt{d_{k}}}\right)V$$
  Here, the scaling factor $\sqrt{d_{k}}$ normalizes the dot products, as mentioned earlier, to avoid extremely large values that could destabilize the softmax function.
3. Concatenation of heads: After each head computes its output, the outputs are concatenated along the feature dimension:
  (3) $$\mathrm{MultiHead}\left(X\right)=\;\mathrm{Concat}\;\left({\mathrm{head}}_{\;1},\;{\mathrm{head}}_{\;2},\ldots ,\;{\mathrm{head}}_{\;h}\right)W^{O}$$
  where ${\mathrm{head}}_{i}=\mathrm{Attention}\left(XW_{i}^{Q},XW_{i}^{K},XW_{i}^{V}\right)$, and $W^{O}$ is another learned weight matrix that combines the outputs from all the heads.
4. Output transformation: Finally, the concatenated output from all the heads is once again linearly transformed by $W^{O}$ to yield the final output of the multi-head attention layer. This output can then be used in subsequent layers of the model.

By employing multi-head attention, the model can focus on information from various representation subspaces at different positions within the sequence [34], thereby gaining a more holistic understanding of the input data. This approach is especially beneficial for complex tasks like target detection in infrared images, where diverse features may be crucial for making precise predictions.

**Figure 3:**
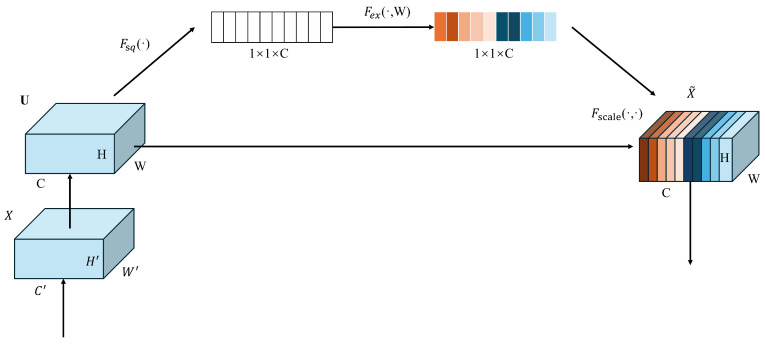
The structure of the SE module.

Attention mechanisms have recently been explored in the field of object detection. Two commonly used attention mechanisms are the SE (Squeeze-and-Excitation) module and the CBAM (Convolutional Block Attention Module). Since these two modules are employed to enhance the YOLOv8n model in our subsequent comparative experiments, we provide a brief introduction to them. The SE attention module is a type of channel attention module that is often used in visual models. It is considered plug-and-play, meaning it can enhance the channel features of the input feature map without altering its size. The “SE” in SE module stands for “Squeeze” and “Excitation”. “Squeeze” refers to the compression of spatial information in the input feature map, while “Excitation” refers to the process of learning channel attention information and combining it with the input feature map to produce a feature map with channel attention. The structure of the SE module is shown in Figure 3:

The SE module primarily consists of two parts: Squeeze and Excitation. The process flow of the module is as follows:

1. Input the feature map: The input feature map has dimensions H × W × C.
2. Spatial feature Squeeze: Compress the spatial features of the input feature map by applying global average pooling across the spatial dimensions, resulting in a feature map of size 1 × 1 × C.
3. Channel feature learning: Learn channel-specific features from the compressed feature map by passing it through a fully connected (FC) layer. This operation produces a feature map with channel attention, maintaining the dimensions 1 × 1 × C
4. Channel attention application: Multiply the channel-attended feature map 1 × 1 × C with the original input feature map H × W × C on a channel-by-channel basis to apply the learned attention weights. The final output is a feature map with enhanced channel attention. In this process, the FC layer predicts the importance of each channel, applying different weights based on the importance of each channel.

**Figure 4:**
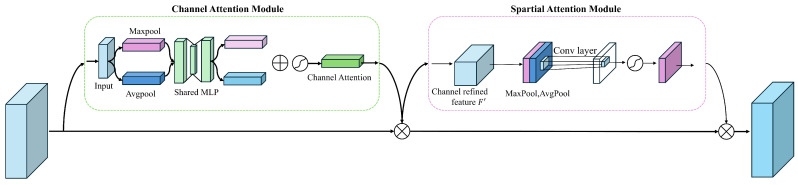
The structure of the CBAM.

CBAM (Convolutional Block Attention Module) can apply attention in both the channel and spatial dimensions. The structure of CBAM is shown in Figure 4:

CBAM consists of two independent submodules: the Channel Attention Module (CAM) and the Spatial Attention Module (SAM), which apply attention in the channel and spatial dimensions, respectively. This not only saves parameters and computational resources but also ensures that CBAM can be integrated as a plug-and-play module into existing network architectures.

In the Channel Attention Module (CAM), the input feature map F(H × W × C) undergoes global max pooling and global average pooling to produce two 1 × 1 × C feature maps, which are then processed by a shared two-layer neural network (MLP) with the first layer having C/r neurons (where r is the reduction ratio) with ReLU activation and the second layer having C neurons; the outputs of the MLP are combined using element-wise addition and passed through a sigmoid activation to generate the final channel attention feature Mc, which is then multiplied element-wise with the input feature map F to produce the input feature for the Spatial Attention Module.

In the Spatial Attention Module (SAM), the output feature map F’ from the Channel Attention Module serves as the input, which undergoes global max pooling and global average pooling to produce two H × W × 1 feature maps, these are concatenated along the channel axis, passed through a 7 × 7 convolution to reduce dimensionality to H × W × 1, followed by a sigmoid activation to generate the spatial attention feature Ms, which is then multiplied with the input feature map to produce the final output feature map.

## 3. Method

### 3.1. DyHead Detection Head Improvement

**Figure 5:**
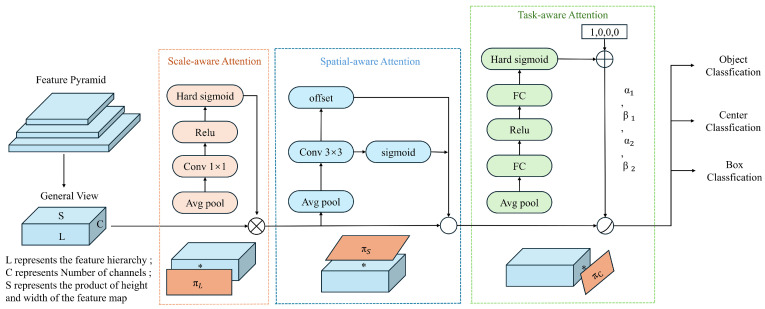
DyHead model framework diagram.

For small object detection, the limited inherent features make it challenging to extract beneficial semantic information during network training. Moreover, repeated downsampling and pooling operations can lead to the loss of a significant amount of small object features, making it difficult for the model to accurately locate and recognize these objects. Therefore, it is necessary to enhance the feature information of small objects without increasing the model’s complexity. Integrating attention mechanisms into the network can help the model focus on more important regions and assign appropriate weights to them, thereby enabling the efficient use of resources. Infrared small targets, in particular, have similar infrared characteristics that can easily blend into the background. Due to the varying scales and spatial locations of these targets, feature extraction and object detection become significantly challenging. To address these issues, this study introduces the DyHead (Dynamic Head) based on a self-attention mechanism, which quickly identifies regions of interest while ignoring distracting information. This helps the model capture the global spatial information of the feature map and enrich the contextual semantic information. DyHead, proposed by Dai et al. [35], is a detection head based on the attention mechanism. It integrates multi-head self-attention mechanisms into scale-aware feature layers, spatial-aware location layers, and task-aware output channels within a unified framework. This integration enhances the detection performance of the detection head without additional computational overhead. As illustrated in Figure 5, the DyHead framework adjusts the scales of features from the original Feature Pyramid Network, restructuring them into a three-dimensional tensor with uniform scale, and employs different attention mechanisms at each independent dimension. Among them, * represents the operation between the feature map and the attention function.

DyHead integrates scale attention, spatial attention, and task attention within a single module, treating the input to the head, referred to as the General View, as a three-dimensional tensor. This tensor consists of *L*, representing different scales (i.e., different layers and stages) of feature maps; *S*, representing spatial location information, which is the product of the width and height of the feature maps; and *C*, representing channel information. DyHead utilizes a decoupled attention mechanism, allowing each dimension to independently engage in feature perception through its attention mechanism. The three types of attention mechanisms are as follows:

- Scale-aware attention is implemented at the level dimension, providing scale-sensitive attention. As different levels of feature maps correspond to different target scales, the introduction of this mechanism enhances the model’s ability to perceive scale variations.
- Spatial-aware attention is implemented at the spatial dimension, providing spatial-sensitive attention. Changes in spatial positioning correspond to geometric transformations of the detection targets, and the introduction of this mechanism enhances the model’s ability to perceive spatial positions.
- Task-aware attention is implemented at the channel dimension, providing task-sensitive attention. Different channels correspond to different detection tasks, and the introduction of this mechanism enhances the model’s ability to perceive and differentiate between various tasks.

To avoid the high computational cost associated with fully connected layers, the dynamic detection head adopts a methodology using three sequential attention mechanisms, as shown in Equation (1). Each attention module corresponds to a single dimension:

(4) $$W\left(F\right)=\pi_{c}\left(\pi_{s}\left(\pi_{l}\left(F\right)\cdot F\right)\cdot F\right)\cdot F$$

Here, $\pi_{l}$ represents the scale-aware attention function, which enhances the perception of different scale features; $\pi_{s}$ is the spatial-aware attention function, which strengthens the model’s ability to perceive spatial locations; and $\pi_{c}$ is the task-aware attention function, which boosts the model’s sensitivity to different task-oriented targets. Adding a dynamic head after the backbone network aims to enhance the model’s adaptability to different target scales and feature hierarchies, thereby improving the detection accuracy and generalization ability of the model.

### 3.2. Gather-and-Distribute Mechanism Improvement

**Figure 6:**
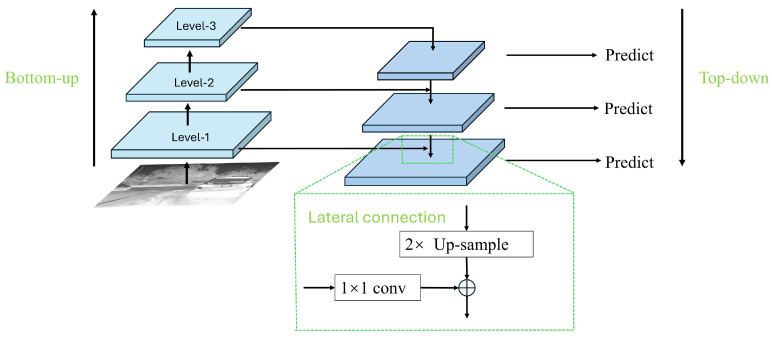
The traditional Feature Pyramid Network (FPN) structure diagram.

In existing general object detection CNN models, multi-scale detection leverages lower-level feature layers to help the model acquire accurate localization information and discriminative feature details, which is beneficial for detecting and recognizing small targets. Employing innovative multi-scale feature fusion techniques can take advantage of the high-resolution information from the lower layers and the strong semantic features from the higher layers, thereby improving the accuracy of small target detection. Since the amount of feature information for small-scale targets is relatively limited, it is crucial to make full use of the detailed information in the images. In the YOLOv8 series, the intermediate layer structure incorporates the traditional Feature Pyramid Network (FPN) architecture, which includes multiple branches for multi-scale feature fusion. The FPN is designed to efficiently combine low-resolution, semantically strong features with high-resolution, semantically weaker features through a top-down pathway and lateral connections. Each level of the pyramid can be used for detecting objects at different scales, which is particularly beneficial in environments where target sizes vary significantly.The FPN structure is shown in Figure 6.

The traditional Feature Pyramid Network (FPN) structure merges features only from adjacent levels, accessing information from other layers in an indirect “recursive” manner. As illustrated in Figure 4, existing Levels 1, 2, and 3 are arranged from top to bottom, with FPN facilitating the fusion between these levels. This transfer method may result in significant information loss within the traditional FPN structure. Inter-layer information exchange is restricted to selected information from intermediary layers, while unselected details are discarded during transmission. Consequently, information at a certain level can only adequately support adjacent layers, reducing the support provided to other global layers. Thus, the overall effectiveness of information fusion could be compromised.

**Figure 7:**
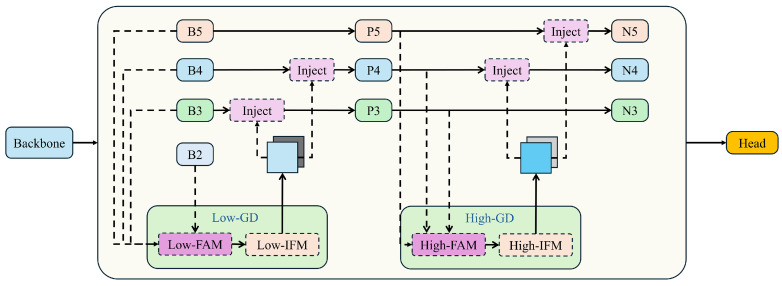
The “Gather-and-Distribute” (GD) mechanism structure diagram.

To reduce information loss during the transmission process in traditional FPN structures, this paper leverages the GD mechanism to improve YOLOv8’s feature fusion algorithm, making it better suited to the challenges of small object detection. Wang et al. proposed a new “Gather-and-Distribute” (GD) mechanism to replace the original recursive method. This mechanism employs a unified module that collects and merges information from all levels and then redistributes it across different levels. In this study, we combine this mechanism with self-attention to optimize the information distribution module. This approach not only prevents the inherent information loss present in traditional FPN structures but also enhances the fusion capability of intermediate layer information without significantly increasing latency. The “Gather-and-Distribute” (GD) mechanism is illustrated in Figure 7.

In the implementation of the Gather-and-Distribute Mechanism, the collection and distribution processes are managed by three key modules: the Feature Alignment Module (FAM), the Information Fusion Module (IFM), and the Information Injection Module (Inject).

The collection process involves two steps. First, the FAM collects and aligns features from different levels. Then, the IFM merges these aligned features to create global information. Once the global information is fused, the Injection module utilizes a simple attention operation to distribute this information to each level, enhancing the detection capabilities of the branches.

To improve the model’s ability to detect objects of various sizes, it incorporates two branches: the Low-Gather-and-Distribute (Low-GD) branch and the High-Gather-and-Distribute (High-GD) branch. These branches, respectively, extract and merge features from large and small size feature maps. Assuming the input image shape is N×3×H×W, and the backbone generates multi-scale features at four levels: B2 $\left(N\times Cb_{2}\times H/4\times W/4\right)$, B3 $\left(N\times Cb_{3}\times H/8\times W/8\right)$, B4 $\left(N\times Cb_{4}\times H/16\times W/16\right)$, and B5 $\left(N\times Cb_{2}\times H/32\times W/32\right)$, where *N* represents the batch size, $Cb_{i}$ the channel count at different scales, and RBi the height and width at different scales.

#### 3.2.1. Low-GD Branch

**Figure 8:**
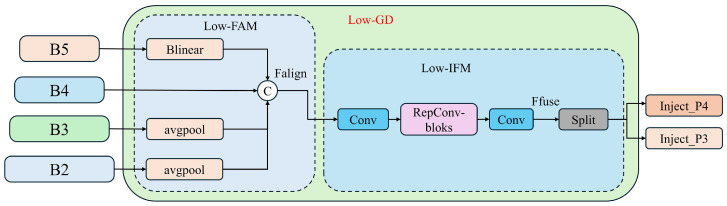
The low-stage Gather-and-Distribute branch structure diagram.

The Low-GD branch, through feature fusion of B2, B3, B4, and B5, yields high-resolution features that retain information about small targets, as depicted in Figure 8.

**Figure 9:**
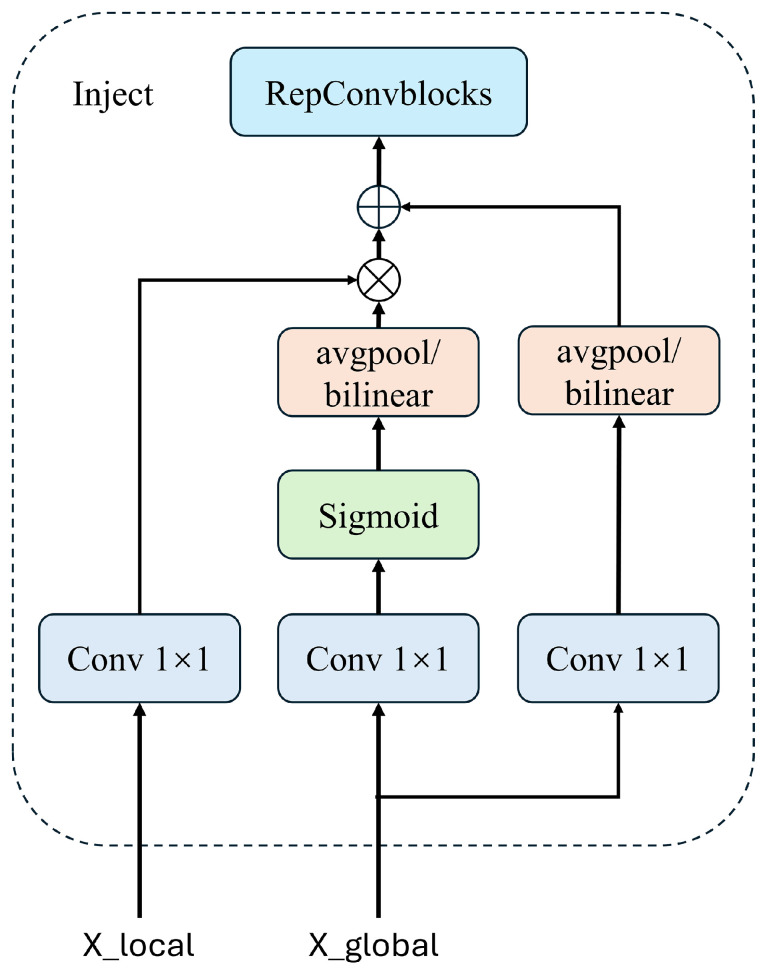
Schematic diagram of self-attention mechanism in information distribution module.

- Low-FAM (Low-level Feature Alignment Module). As depicted in the diagram, B4 serves as the reference for alignment. Larger feature maps such as B2 and B3 are downsampled through average pooling, while smaller feature maps like B5 are upsampled using bilinear interpolation to standardize the size of the feature maps. The aligned feature maps are then concatenated to form the merged feature $F_{align}$$\left(N\times C_{concat}\times H/8\times W/8\right)$. This is followed by processing through a Transformer module, which minimizes computational complexity.
- Low-IFM (Low-level Information Fusion Module). The design of this module includes a Convolution (Conv) module, RepBlock module, and a Split operation. The aligned and concatenated features $F_{align}$ are input into the RepBlock module to obtain the fused features $F_{fuse}$. The Conv module adjusts the channel dimensions to accommodate different model sizes. $F_{fuse}$ is then split along the channel dimension into $F_{inj\_P3}$ and $F_{inj\_P4}$, which are used for subsequent feature fusion with different layers. The equations are as follows:
  (5) $$F_{align}=\mathrm{Low-FAM}\left(\left[B_{2},B_{3},B_{4},B_{5}\right]\right)$$
  (6) $$F_{fuse}=\mathrm{RepBlock}\left(F_{align}\right)$$
  (7) $$\left(F_{inj\_P3},F_{inj\_P4}\right)=\mathrm{Split}\left(F_{fuse}\right)$$
- Inject (Information Distribution Module). The distribution of information is facilitated using a self-attention mechanism, as illustrated in Figure 9. This approach ensures that each layer receives the most relevant and contextually appropriate information, enhancing the model’s overall detection capability.

The inputs for the information distribution module include the local features at the current scale, $x_{\mathrm{local}}$ (denoted as $F_{\mathrm{local}}$, such as B3 and B4), and the global features generated by the Information Fusion Module (IFM), denoted as $X_{\mathrm{global}}$ (such as $F_{\mathrm{inj\_P3}}$ and $F_{\mathrm{inj\_P4}}$). If there is a size mismatch during the fusion process, it can be addressed through methods like average pooling or bilinear interpolation to standardize the feature sizes. The features are then further extracted and fused through a RepBlock. The equations for these processes are as follows:

(8) $$F_{\mathrm{global\_act\_Pi}}=\mathrm{resize}\left(\mathrm{Sigmoid}({\mathrm{Conv}}_{\mathrm{act}}\left(F_{\mathrm{inj\_Pi}}\right))\right)$$

(9) $$F_{\mathrm{global\_embed\_Pi}}=\mathrm{resize}\left({\mathrm{Conv}}_{\mathrm{global\_embed\_Pi}}\left(F_{\mathrm{inj\_Pi}}\right)\right)$$

(10) $$F_{\mathrm{att\_fuse\_Pi}}={\mathrm{Conv}}_{\mathrm{loca\_embed\_Pi}}\left(B_{i}\right)\times F_{\mathrm{global\_act\_Pi}}+F_{\mathrm{global\_embed\_Pi}}$$

(11) $$P_{i}=\mathrm{RepBlock}\left(F_{\mathrm{att\_fuse\_Pi}}\right)$$

This sequence of operations ensures that both local and global features are effectively integrated, enhancing the detection capabilities of the model by effectively leveraging both the detailed local context and the broader contextual insights provided by the global features. This method not only ensures consistency in feature dimensions across different levels but also enhances the model’s ability to recognize and respond to various object scales and complexities effectively.

#### 3.2.2. High-GD Branch

**Figure 10:**
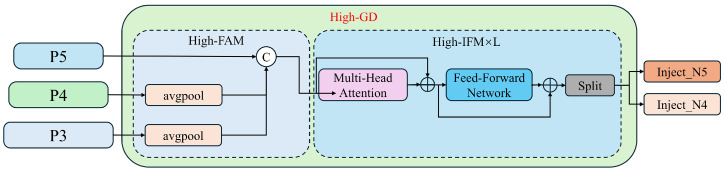
The high-stage Gather-and-Distribute branch structure diagram.

Features P3, P4, and P5, obtained through Low-GD fusion, are further processed using High-GD for feature fusion, as illustrated in Figure 10:

(1) High-FAM (High-Order Feature Alignment Module): This module consists of an average pooling layer (AvgPool), which reduces the dimensions (width and height) of the input features to a uniform size. Initially, the dimensions of P3 and P4 are reduced to match those of P5, followed by merging using the Concat operation.

(2) High-IFM (High-Order Feature Integration Module): This module primarily comprises a Transformer and a Split operation. The steps are outlined as follows: The feature $F_{\mathrm{align}}$ obtained from the FAM first passes through the Transformer to yield $F_{\mathrm{fuse}}$. A 1×1 convolution adjusts the channels of $F_{\mathrm{fuse}}$ to $C_{P4}+C_{P5}$, facilitating subsequent Split operations. The feature map is then segmented into $F_{\mathrm{inj\_N4}}$ and $F_{\mathrm{inj\_N5}}$ through the Split operation.

The process formula is as follows:

(12) $$F_{\mathrm{align}}=\mathrm{High\_FAM}\left(\left[P_{3},P_{4},P_{5}\right]\right)$$

(13) $$F_{\mathrm{fuse}}=\mathrm{Transformer}\left(F_{\mathrm{align}}\right)$$

(14) $$F_{\mathrm{inj\_N4}},F_{\mathrm{inj\_N5}}=\mathrm{Split}\left(\mathrm{Conv}1\times 1\right)$$

The Transformer fusion module in Equation (8) consists of multiple stacked Transformer blocks, denoted by L, each including a multi-head attention block, a feed-forward network (FFN), and residual connections.

(3) Information Distribution Module: The distribution modules for High-GD and Low-GD are identical.

(15) $$F_{{\mathrm{global\_act\_N}}_{i}}=\mathrm{resize}\left(\mathrm{Sigmoid}\left({\mathrm{Conv}}_{\mathrm{act}}\left(F_{{\mathrm{inj\_N}}_{i}}\right)\right)\right)$$

(16) $$F_{{\mathrm{global\_embed\_N}}_{i}}=\mathrm{resize}\left({\mathrm{Conv}}_{{\mathrm{global\_embed\_N}}_{i}}\left(F_{{\mathrm{inj\_N}}_{i}}\right)\right)$$

(17) $$F_{{\mathrm{att\_fuse\_N}}_{i}}={\mathrm{Conv}}_{{\mathrm{loca\_embed\_N}}_{i}}\left(P_{i}\right)\cdot F_{{\mathrm{global\_act\_N}}_{i}}+F_{{\mathrm{global\_embed\_N}}_{i}}$$

(18) $$N_{i}=\mathrm{RepBlock}\left(F_{{\mathrm{att\_fuse\_N}}_{i}}\right)$$

### 3.3. Model Pruning

In convolutional neural networks, feature mapping between channels may exhibit redundancy, making it feasible to apply direct pruning based on the magnitudes of the weights [36]. For channel layers, model reconstruction can be pursued using a method that minimizes feature errors. Initially, weight magnitudes are used as criteria for sparsification to identify the most representative channels. Subsequently, the remaining redundant channels are trimmed, thereby refining the model to enhance its accuracy and performance. Iterative pruning may also be employed to remove the majority of redundant channels, eliminate errors, and improve model precision.

**Figure 11:**
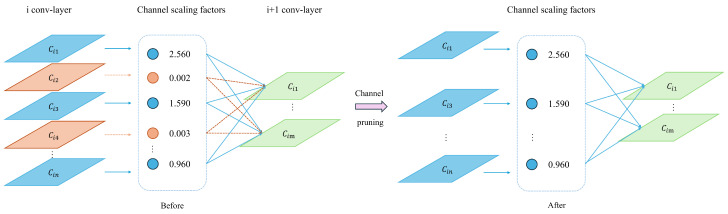
Schematic diagram of channel trimming.

Structured pruning techniques excel in complex convolutional neural networks by directly trimming entire channel layers or convolutional kernels, optimizing the model. This approach reduces computational demand by cutting parameters, thereby directly accelerating the inference speed of the convolutional neural network models. It transforms originally complex networks into simpler ones, ultimately achieving network light-weighting. This article mainly uses the channel pruning technique shown in Figure 11.

Channel pruning fundamentally reduces the model size and the number of parameters, thereby decreasing the actual computational workload. In channel pruning techniques, the γ coefficients of the Batch Normalization (BN) layers are utilized to assess the contribution score of each channel. The diagram describes the process of the channel pruning algorithm. Equations (19)–(22) detail the computation methodology in the BN layer.

(19) $$\mu =\frac{1}{m}\sum_{i=1}^{m}x_{i}$$

(20) $$\sigma^{2}=\frac{1}{m}\sum_{i=1}^{m}x_{i}-\mu^{2}$$

(21) $${\hat{x}}_{i}=\frac{x_{i}-\mu }{\sqrt{\sigma^{2}+\varepsilon }}$$

(22) $$y_{i}=\gamma {\hat{x}}_{i}+\beta \equiv BN_{\gamma }x_{i}$$

In Equations (8) to (11), γ and β represent small-batch learnable parameters. Based on the distribution of the γ coefficients and the pruning rate of the channel pruning algorithm, channels with high contributions (displayed in blue) are retained, while those with low contributions (displayed in orange) are eliminated. When forming connections, neurons from channels with lower contributions are not involved.The specific steps for channel pruning are as follows:

1. Pretrain: Pretraining involves regular training procedures to develop a model that is subsequently subjected to constrained training.
2. Constrained training: To promote structural sparsity within the YOLOv8 infrared small target detection model, L1 regularization is applied to the coefficients of the Batch Normalization (BN) layers. Constrained training facilitates easier pruning by allowing the model to identify channels or weight coefficients that are less important. These elements are then targeted in the pruning process to achieve model compression. Direct pruning without this preliminary step might lead to significant issues such as a drastic reduction in model accuracy and uneven pruning. Therefore, sparse training before pruning enables more precise identification of expendable channels or weights, thus preventing such problems.
3. Pruning: After sparse training, pruning is performed according to a predefined rate to create a compact model that requires less storage capacity.
4. Model fine-tuning: The primary goal of fine-tuning is to effectively recover the accuracy that might be compromised due to significant losses caused by channel pruning.

This study also extensively investigates the appropriate pruning levels and model configurations specifically tailored for the task of infrared small target detection. Throughout the research, we evaluate the trade-offs between performance degradation, parameter reduction, and computational complexity. Through this analysis, a pruning retention rate of 50% is identified as the optimal value.

## 4. Experiment

### 4.1. Model Evaluation Metrics

To evaluate the detection performance of the model, three metrics are employed: accuracy, recall, and mean average precision (mAP). High accuracy indicates that most detected objects are indeed the targets, while high recall suggests that the model effectively identifies more target objects within the images [37]. At first glance, accuracy and recall might seem sufficient for assessing object detection quality. However, since each image in object detection tasks can contain targets from various categories, distinct metrics are required to evaluate both the classification and localization aspects of the model [38]. mAP is widely used in object detection as a measure of recognition precision, representing the area under the precision–recall (*P*-*R*) curve. The formulas for calculating mAP, precision (*P*), and recall (*R*) are as follows:

- Precision (*P*): This is the ratio of correctly predicted positive observations to the total predicted positives. It is calculated as:
  (23) $$P=\frac{TP}{TP+FP}$$
  where TP is the number of true positives, and FP is the number of false positives.
- Recall (*R*): This is the ratio of correctly predicted positive observations to the all observations in actual class. It is defined as:
  (24) $$R=\frac{TP}{TP+FN}$$
  where FN is the number of false negatives.
- Mean average precision (mAP): For multiple classes, mAP is the mean of the average precision calculated for each class. Average precision (AP) for a single class is the area under the precision–recall curve for that class. The mAP across all classes is calculated by averaging the APs:
  (25) $$\mathrm{mAP}=\frac{1}{N}\sum_{i=1}^{N}AP_{i}$$
  where *N* is the number of classes, and $AP_{i}$ is the average precision for class *i*.

These metrics provide a comprehensive evaluation of the model’s detection accuracy, its ability to detect all relevant objects, and its overall precision–recall performance across different object categories.

### 4.2. Dataset Creation and Experiments

The dataset used in this study adopts a hybrid approach that combines network scraping and public dataset integration. We have selected a large number of road infrared images, featuring small targets such as pedestrians and vehicles. To improve the generalizability of our results, we selected images from different times of the day, weather conditions, and lighting conditions. Among them, there were about 2215 images captured by the network. After data cleaning, duplicate and low resolution images were filtered out, and finally 2032 images remained. After using Labelimg for image annotation and mixing it with a public dataset, a total of 8216 images were used for training, each containing multiple random infrared targets (cars or people). The dataset is divided into training set, validation set, and testing set in a ratio of 7:2:1.

**Figure 12:**
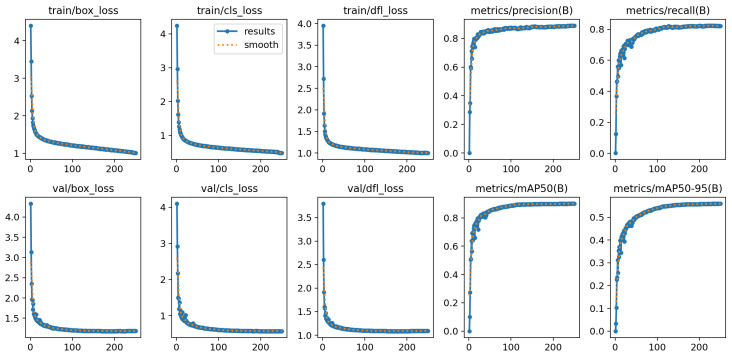
Improved model training result graph.

An improved network model was developed using the PyTorch open-source deep learning framework. Following development, the model underwent training and object detection experiments. The hardware used included an Intel Core i7-13700KF CPU (Intel, Santa Clara, CA, USA) and an NVIDIA Geforce RTX 4090 GPU (NVIDIA, Santa Clara, CA, USA). The batch size was set to 64, and the base learning rate was 0.008. In RTAL, α was set to 1 and β to 6. The weights for the classification, CIOU, and DFL loss functions were set to 1.0, 2.0, and 0.05, respectively. Training acceleration was achieved using CUDA 11.2 and CUNN 8.2. For testing, the same Intel Core i7-10700 CPU (Intel, Santa Clara, CA, USA) used in the training phase was employed to predict small infrared targets. The training outcomes are illustrated in Figure 12.

To account for model size, this study also employs model size comparison as metrics for evaluating computational speed. We used YOLOv8n as the baseline model and compared it with versions enhanced by the SE (Squeeze-and-Excitation) attention mechanism, the CBAM (Convolutional Block Attention Module) attention mechanism, as well as the improved model proposed in this study, MSSA (multi-scale self-attention mechanism). The results related to these metrics are shown in Table 1. It is important to note that, to ensure a fair comparison in the experiments, the model in this case did not undergo any prune operations.

**Figure 13:**
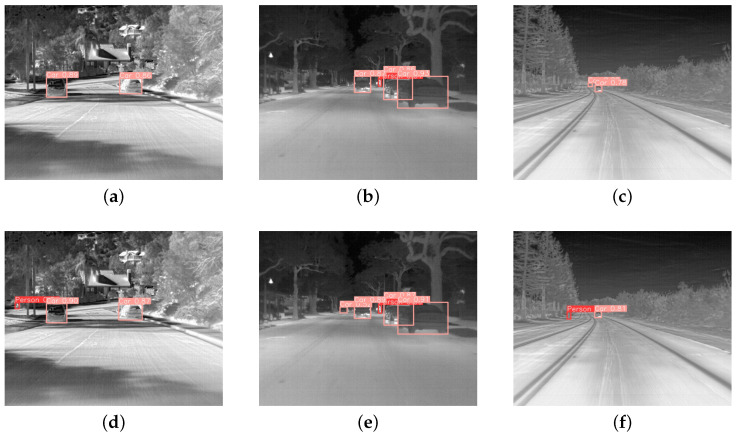
The detection results of different algorithms. (**a**–**c**) are the detection results of YOLOv8 improved by SE attention, and (**d**–**f**) are the detection effect diagrams of the improved model in this paper.

Table 1 reveals that the improved YOLOv8 in this study achieves higher precision–recall rates and mean average precision (mAP) compared to other object detection algorithms. The other related metrics are also superior to the three alternative methods. Additionally, the pruning-based lightweight operation significantly reduces the model’s parameter count, making the improved YOLOv8 easier to deploy on devices with limited computational power, while still enabling fast and accurate detection of targets. Target detection using the aforementioned five algorithms on an infrared small target dataset is depicted in Figure 13.

As shown in Figure 13, our improved algorithm demonstrates a significantly higher recall rate compared to the algorithm enhanced with SE attention mechanism, accurately detecting small distant targets such as pedestrians and vehicles. This is crucial for the safety in areas like autonomous driving, as it directly impacts the vehicle’s ability to make timely decisions, such as deceleration, at a safe distance. Similar to YOLOv8n-SE, our algorithm integrates an attention mechanisms into the model. However, unlike YOLOv8n-SE, which applies the SE module only to compute multi-scale feature blocks on the intermediate layers of the network, our approach has some distinct advantages. In YOLOv8n-SE, the learned feature distribution may not necessarily align with the pixel distribution assumptions for small infrared targets in the original image, leading to limited performance improvements.

In contrast, our algorithm calculates self-attention mechanisms across multiple scales of the original image and uses the GD module for feature fusion. The learned feature distribution aligns more closely with the pixel distribution of small targets in the original image. By embedding this into the target detection network, we can expand the spatial distribution of small targets, enhance weak targets, effectively suppress background interference, and reduce loss during feature transmission. Additionally, the DyHead module is introduced in the network prediction head to capture global attention, further improving the detection performance of small infrared targets.

To validate the superiority of the proposed object detection model, we conducted verification tests on the MMDetection platform. MMDetection is an open-source project launched by SenseTime and the Chinese University of Hong Kong for object detection tasks. It implements numerous object detection algorithms based on PyTorch, encapsulating processes such as dataset construction, model development, and training strategies into modules. The comparison results of our proposed model with other object detection algorithms on the MMDetection platform are summarized in Table 2.

Through comparative experiments, it can be seen that our improved model performs well on our self-built dataset compared to other methods, with good improvements in precision and recall metrics.

### 4.3. Ablation Study

To validate the efficacy of the improvements proposed in this paper, ablation experiments were conducted. These experiments specifically addressed the integration of the self-attention mechanism within the DyHead module (abbreviated as DyHead), the introduction of the G-D (Guided Distillation) mechanism in the Neck part of the network (abbreviated as GD), and the effects of pruning operations. The ablation experimental indicators are shown in Table 3.

As shown in Table 3, the improved self attention module was used to replace the original C2f module, and combined with the G-D mechanism that also utilizes self attention, the mAP of the model was effectively enhanced at an IoU threshold of 0.5 to 0.95. Additionally, the use of pruning operations significantly reduced the model’s parameter count and overall size. This not only lightens the model but also greatly enhances the model’s deployment and generalization capabilities across various platforms, making it more versatile and efficient in real-world applications. This ablation study highlights the contribution of each individual component to the overall performance improvement of the model, substantiating the modifications as beneficial for enhancing detection accuracy while optimizing computational resources.

### 4.4. Target Heatmaps and Detection Effects

In computer vision, heatmaps are a visualization technique used to represent the intensity or significance of specific attributes or features within an image [39]. Typically, heatmaps are displayed using a color gradient, where variations in color indicate changes in data magnitude or intensity. These maps often use a spectrum from cool to warm colors, such as blue to red, with red usually denoting higher values or greater areas of interest and blue representing lower values or lesser focus.

In neural networks that utilize attention mechanisms, heatmaps can help visualize the areas of an image the model focuses on during processing. Additionally, heatmaps can illustrate the spatial support regions for image classification decisions, highlighting which parts of the image contribute most significantly to the classification results.

This paper utilizes Gradient-weighted Class Activation Mapping (Grad-CAM) [40], a popular computer vision technique, to generate heatmaps that visualize the areas of focus for convolutional neural networks during image classification specific to certain categories. Grad-CAM produces localized heatmaps for specific categories by weighting the feature maps with the gradient of the target class’s output scores. Specifically, this technique focuses on a particular convolutional layer within the network, utilizing the feature maps from that layer and the gradient information of these maps with respect to a class output. This method is primarily used to explain the decision-making process of deep learning models, particularly in understanding how the model responds to specific areas of different inputs.

**Figure 14:**
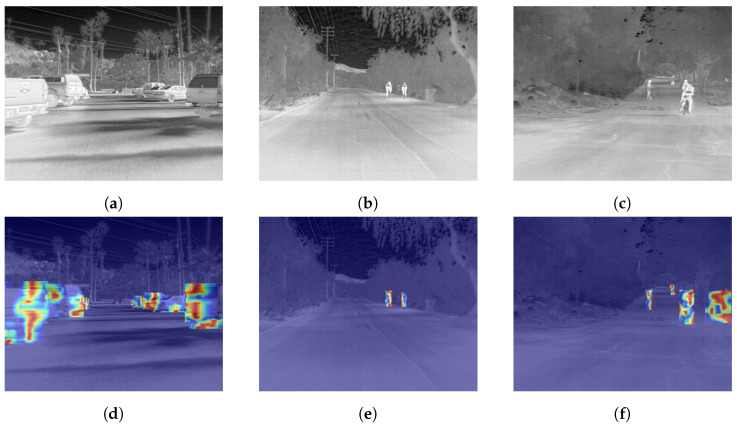
The detection results of the heatmap, where (**a**–**c**) are the original images, and (**d**–**f**) are the improved model detection effect maps.

The heatmaps from the improved model demonstrate how attention mechanisms enhance the model’s ability to focus on relevant features, significantly impacting classification accuracy. The visualization of these effects, as shown in Figure 14, offers insights into the spatial aspects of the model’s decision-making process, highlighting the regions within the images that contribute most to the detected categories.

**Figure 15:**
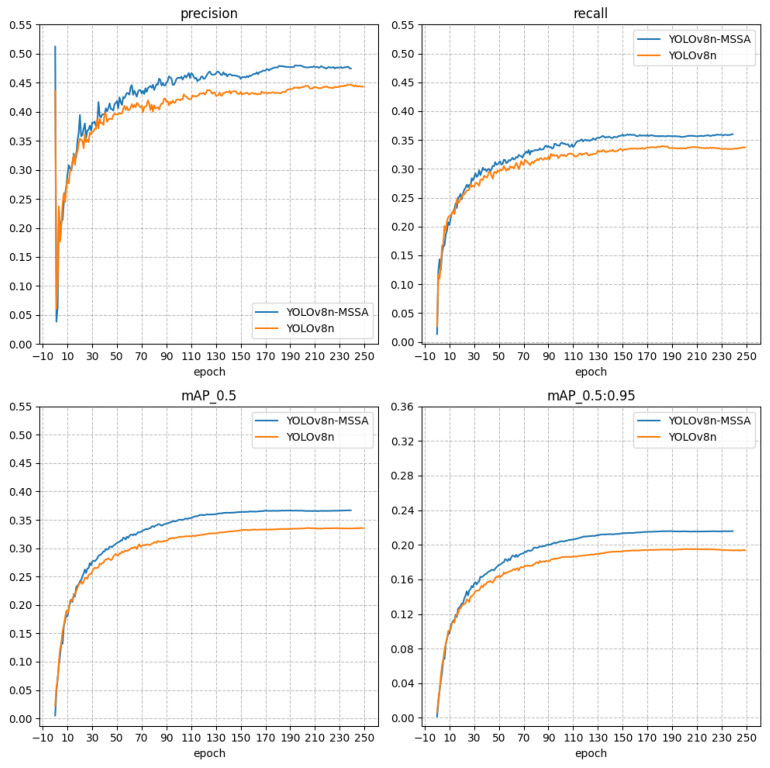
Experimental results on the VisDrone2019 dataset.

To evaluate the generalization capability of the proposed model, it was tested on the VisDrone2019 dataset [41] and compared with the baseline YOLOv8n model. The VisDrone2019 dataset, compiled by the AISKYEYE team at the Machine Learning and Data Mining Lab of Tianjin University, mainly consists of 12 types of targets to be detected. The experimental results, displayed in Figure 15, show that our model, abbreviated as MSSA (multi-scale self-attention), demonstrates a 3.1% improvement in AP and a 2.5% increase in mAP on the VisDrone2019 dataset compared to the original YOLOv8n model. This improvement in performance underscores the model’s enhanced generalization ability, making it suitable for applications in small target detection across various fields. The MSSA model’s success in this challenging dataset highlights its potential effectiveness and adaptability in real-world scenarios where small object detection is crucial.

## 5. Conclusions

This paper presents an enhanced YOLO object detection algorithm, leveraging a multi-scale self-attention mechanism to detect small road objects in infrared detection scenes. The key innovation lies in integrating the robust multi-scale information fusion capabilities of the self-attention mechanism with the real-time performance of the YOLO algorithm. This integration facilitates rapid and accurate detection and recognition of small objects in infrared images. To validate the effectiveness of the proposed model, extensive experiments were conducted using publicly available infrared image datasets. These datasets feature infrared images captured in diverse complex environments, enabling effective testing of the method’s detection and recognition capabilities across different settings.

The experimental results indicate significant improvements in detection speed and recognition accuracy over traditional detection methods. The research achieves swift detection and recognition of small infrared targets, offering a potent solution that contributes importantly to the development and practical application of autonomous driving technology. However, the study also acknowledges certain limitations, such as potential shortages of computational resources when processing large volumes of image data and the need for enhanced accuracy in recognizing small targets in complex environments. Future research will focus on further refining our approach to provide even more effective solutions.

## Figures and Tables

**Table 1:** Comparison results of different attention mechanisms.

Model	Precision%	Recall%	mAP@0.5/%	mAP@0.5:0.95/%	Params/M
YOLOv8n	88.5	80.2	90.1	52.5	3.2
YOLOv8n-CBAM	89.2	81.3	91.4	54	3.9
YOLOv8-SE	89.3	82.1	91.6	53.9	3.7
YOLOv8-MSSA *	89.5	82	92.3	55	4.0

* The model here has not undergone any pruning operation.

**Table 2:** Algorithm comparison indicators.

Model	Precision/%	Recall/%	mAP@0.5/%	mAP@0.95/%
YOLOv8n (base)	87.5	80.2	89.4	53.5
SSD	85	77.8	87.9	52
MASK_RCNN	87	79	89.5	53.2
Faster-RCNN	85.2	78.9	89.2	52.9
YOLOv8n-MSSA * (ours)	89.5	82	90	55.9

* The model here has not undergone any pruning operation.

**Table 3:** The ablation experimental indicators.

Baseline	DyHead	GD	Prune	Precision/%	Recall/%	mAP @0.5/%	Params/M
✓	×	×	×	88.5	80.2	90.1	3.2
✓	✓	×	×	89.2	80.1	91.5	3.6
✓	×	✓	×	89	81.5	92.2	3.9
✓	×	×	✓	88.3	79.8	89.8	2.3
✓	✓	✓	✓	89.3	81.9	92	2.5

## Data Availability

Data are contained within the article.
